# The Classes and the Masses: A Word to the Wise

**Published:** 1886-12-25

**Authors:** 


### The Classes and the Masses: a Word to the Wise.

By the Ven. Archdeacon Farrar, D.D.

### II.—A CHRISTMAS APPEAL: HELP THE HOSPITALS.

The Work of the Good Physician.

And this was his example: "He went about doing good." For thirty years, indeed, He lived as the carpenter of Nazareth; lived as we have all to live, surrounded by common human influences, engaged in common daily duties, earning bread by toil of brain or sweat of brow, with mother, brethren, and sisters in His village home. This He did to show us that there is nothing unsacred, nothing ignoble, in our common daily duties, the anxieties, the domesticity, the ordinary surroundings. Not otherwise could He have revealed to us the dignity of man simply as man; the sacredness of home, be it never so homely; the possibility, by pureness and kindness, of living even in the lowliest hovel as a son of God. But when His life burst into the consummate glory of His ministry, then "He went about doing good." Where do you find Him? You will not find Him in some deep seclusion meditating over an open grave; or sitting, with a skull before Him, beating His breast with a stone in a hermit's cavern; or elaborating minutiæ of legalism in some school of the rabbis; or absorbed in ritualism among bands of sacrificing priests. No; but you will find Him everywhere in the bright, busy, varied, suffering, common life of a man with men; attending their feasts and marriages, smiling with their laughter, weeping amid their tears, teaching in their synagogues, healing the sick, cleansing the leper, releasing the tortured soul of the demoniac, restoring to father and mother and sister their much-loved dead. You will find Him taking the little children up in His arms and laying His hands upon them and blessing them. You will find Him giving sight to the blind, and hearing to the deaf, and consolation to the sorrowful, and self-respect to the despised, and love to the hated, and to the most fallen the conviction that there might yet be joy in heaven for them. You will find Him doing the work of the Good Physician for men's bodies as well as for their souls. And if there be one scene which strikes the first three evangelists alike as one which most sets forth His glory, it was that evening when, while the rosy sunset fell on the silver lake, the stillness of the Sabbath twilight was broken as the people thronged to Him with their demoniacs and their diseased. The groans and sighs of that collective misery touched His inmost soul. He suffered with those whom He saw suffer; He sighed for them; His heart bled for them; He bore their griefs; He carried their sorrows; and as He moved amongst them in His love and tenderness, His heart was thrilled with a more than human rapture, as He laid on each unhappy and tortured sufferer the touch of His healing hand.

The Lesson of Christ's Life.

Yes, sympathy, not seclusion; beneficence, not asceticism; mercy, not sacrifice; love, not Pharisaism. There, for those who care to learn it, is the secret of the life of Christ. He went about doing good. And what is the lesson for us? What is it which Christ's voice says to us across the centuries? It says to us, You are living in a world of sin and sorrow. The sorrow touches you all that you may learn a common sympathy. You may shut out for a time—yea, for years,—in slothful luxury and in selfish isolation, the still, sad music of humanity, the moan of its anguish, the cry of its oppression. You may live for yourself, not for others, not for God. The worse for you. You cannot escape from the solidarity which makes you one of a fallen and ruined race. You cannot shut yourself up in some sweet circle of the orthodox, or of the elect, and give to your selfish spleen the name of religion. Old age will steal upon you. Pain will lay upon you her fiery finger. The pale, chilly shadow of death will glide into your houses. And thereby the Lord bids you leave your callous-hearted worldliness, or your cliques of selfish religionism, and take your part, as He did, in the suffering, struggling world. He came to enlighten its errors, to break the yoke of its oppressors, to lull its pangs with an everlasting anodyne, to give to its living and its dying an immortal hope. He came to send into the ravage and the misery ten thousand white-winged messengers of mercy, to minister at least to the anguish they cannot extinguish, and to move, like angels, in the midst of it, with hearts of compassion and words of healing love.

Moral Suicides.

Go to any of our great hospitals. Look at that poor little Lazarus suffering from a complication of leprosies to which he was born. What made him so? The will of God? No! but the vice of his parents. Look at that poor bruised woman felled last night in one of your licensed ginshops by the foul hand of the brute which she calls her husband. What reduced her to that condition? The will of God? No! but the curse of drink. Look at that case of delirium tremens, in all its revolting ghastliness. Who reduced the man to this heat of wretchedness? God? No! but his own depravity. Look at that young man yonder, dying from the awful Nemesis of his own early vices, his loins full of a sore disease which shall lie down with him in the grave. Who slew all these? God? Nay! but they died of moral suicide, the wilful defiance of God's merciful and beneficent laws.

While it is our duty to mitigate the awful results,

Man's Duty. have we no duty also as regards the awful causes? Even mere sanitary duties are potent against the multiplication of disease. Give the people pure air and pure water, and the atmosphere of cities will not be winnowed by the foul wings of the pestilence. Bring greater cleanliness into homes; and before its touch, as before the touch of Christ, fever and cholera will vanish, as the "black death" and the leprosy have already fled. But much more; help men to live in temperance, soberness, and chastity; and the legion of evil spirits who batten upon human degradation will sicken and die. To heal disease is Christ's work, but to pull down rotting streets, to provide proper dwellings for the poor, to minimise cruel temptations, to train children in habits of self-control, to make it easy to do right and difficult to do wrong, to treat vice and foulness as spectres to be exorcised, as monsters to be expelled,—that, too, is the work of every Christian. By so doing we advance the Kingdom of God. If the task seem hopeless, it is only because we are slothful and selfish. When men become faithful, fearless, loving, Utopia will be but another name for time.

But that bright day will not be yet. For many a long year, while, step by step, we try to remedy, we must also try to heal. That is the work of these our great hospitals, with their clean bright wards, their faithful medical staff, their devoted nurses, their loving Christian visitors. I know no institutions which the Lord Christ would have more deeply loved. Not a day passes but deeds are done in them which He would have approved more than 20,000 daily services, more than 500 fasting communions, if they bring forth no fruit of love. To provide the poor worn mother with a room where, skilfully tended and lovingly cared for, she may recover or die in peace; to surround the poor workman, whose limb has been shattered, with such trained ministrations as shall save him from becoming a helpless burden on those whom he best loves; to help the poor lad, maimed by some accident, to bear more easily the anguish of a boyhood which has thus been blighted; to re-illumine the dulled light in the eyes of children, and on their cheeks the faded rose; and where it is beyond us to give back life's lost sunlight to poor sufferers, at least to tinge with the moonlight colourings of resignation the clouds which have darkened our human lives;—this truly is an angel's, this truly is a Christ-like work. And it is the work of hospitals. We are asked to-day to support them. If we are beneath every appeal but that of selfishness; even on selfish grounds we ought to help them liberally. For if their direct ministrations are mainly for the poor, their resultant blessings flow largely back upon every class alike. It is in them that our surgeons and physicians learn the art of healing. It is in them that our nurses acquire their skill, and pass cheerfully from the dying bed of a great noble to the dying bed of some poor sufferer in Clare-market or Drury Lane. It is through them that there has been so vast a diminution of the annual mortality. It was in them that the use of anæsthetics was perfected which exempts our minds from some of their deadliest terrors. It was in them that the antiseptic treatment was developed, which has already saved thousands of lives. It was in them that men have learnt how cholera is caused and typhoid multiplied. If you have done anything for them, you have been superabundantly rewarded. Out of 4,000,000 inhabitants of London, 1,000,000 directly received relief from the hospitals during this year alone.

The Christ-like Work of the Hospitals.

I would entreat you, then, to regard the opportunity of this season of alms-giving not as a burden, but as a privilege. How very few, alas! of our countrymen are taking any direct personal part in works of good. Ask yourselves, each of you, what you are thus directly doing to alleviate the misery, to elevate the condition, of your brother-men. Multitudes of you, if you be honest, will be forced to admit, "We are doing just nothing outside the circle of our families and the routine by which we earn our daily bread." Well, then, if that be so, you are doubly bound: you are bound by every law, human and Divine, to give to the utmost of your power. Do any of you think that London in this matter is commendably generous? If I am to speak truth, not flattery, I say shamefully the reverse. This wealthiest city in the world—this city, of which the wealth is said annually to increase by £20,000,000—is appealed to by the united voices of every single religious denomination on one Sunday, and it only gives some £40,000—*i.e.*, some seven-hundredth fraction, not by any means of its income, but of its yearly accumulation. Out of our 4,000,000 people, one in every four is directly assisted, and only one in every thousand separately contributes. All classes are to blame. Myriads of the poor, who receive the noble beneficence of hospitals, do not spare for them, all their lives long, so much as the value of one quart of ale from their monstrous luxury of drink.

Not a Burden, but a Privilege.

And the rich—what shall I say of the rich? I say that there are scores of men in London and throughout the country who could save our hospitals from anxiety almost without feeling it. Look at very recent art sales—£2,000 for one dessert service; £1,500 for two flower-pots; £3,000 for a chimney ornament; £10,000 for two rose-coloured vases; £300 for a single lady's dress; £1,000 for the flowers of a single ball. I do not criticise this expenditure; I only say that, if there be such a Pactolus of wealth for these gewgaws of silk and of clay, can

What shall I say of the Rich?

there be by comparison only a drop or two to heal the bodies, to ameliorate the souls of men? Why should the runnel of Charity dribble on as it does, while the full tide of Luxury is still at flood?

"All Classes are to Blame!"

I say all classes are to blame. The working classes, whom it is the abject fashion of the day to fool and to flatter to the top of their bent, are every bit as selfish and every bit as extravagant in their way as the rich are in theirs; and, if it comes to that, the day labourer or the shoeblack, who starves his children and leaves his wife in rags in order to besot his own degradation with beer and gin, is infinitely more guilty than the millionaire who indulges his taste for jewels or bric-à-brac. I should like to know how many thousands of pounds exchange hands every Derby Day, not only among the rich, but even among school-boys, shop-boys, and clerks, and loafing idlers in back slums, who are so ignorant of the whole matter as to be every blackleg's ready dupes. If all this flood of money is forthcoming for the Satanically senseless folly of gambling, is there so little by comparison for worthy and noble purposes? I said that 1,000,000 persons are every year relieved and aided by our London hospitals, and that is a considerable under-statement. Well, if only this million thus directly benefited would but contribute 1*s.* apiece on this Christmas Day, 1886, in gratitude for medical skill, more precious than a king could have purchased a hundred years ago, that shilling apiece would at once produce £50,000, which would not only exceed, but almost double, what London has annually given for her 100 hospitals. Yet, obviously, these shillings are not forthcoming. Were there not ten cleansed? But where are the nine?

Follow in the Blessed Ministries of Mercy.

The case this year is urgent. These blessed institutions in London alone are at this moment £40,000 in debt. Might not, then, some of you who are considering this Christmastide what to give—might you not, with no real self-denial to yourselves—might you, not most conscientiously give gold or a cheque? Might not some of you make for once an infinitely small sacrifice for Christ, and give double or treble or decuple the value of your usual contributions? Will none of you try to-day (were it but for once), with the poor entrusted mammon, to earn something of the true eternal wealth? Do this, my friends, and if you do it in a right spirit, and if you dispatch your gifts to the hospitals of your choice, with a deeper desire to follow, in the blessed ministries of mercy, the example of your Saviour Christ, I am sure that your hearts will be all the happier, and your thoughts all the sweeter, when you lay your heads upon your pillows to sleep to-night.

Who will not Help?

I ask you, then, to do it; I appeal to you all, to the rich, the middle class, the poor, the generous-hearted Americans, and strangers, spending Christmas in England. My voice is feeble and insignificant, but may you not hear, even in my voice, the millionfold reverberation of countless pleading agonies,—yea, the cry of Charity herself, the voice of your Saviour Christ? To you who know what sickness is, I appeal by the sad fellowship of human pain; to you who know it not, I appeal by your gratitude to God for the priceless boon of health, by the sacred name of those who suffer, by the awful mystery of human anguish, by the withered strength and the throbbing nerve, by your own dear children whom you shelter so tenderly, by those other little ones, the shorn lambs of Christ's flock, by the strong man's agony, by the brave man's tears, by the hectic flush upon the young man's cheek, by all the sacredness of human sympathy,—ay, and far more in the name of Him, who dropped the awful plummet of his Godhead into the fathomless abyss of human woe and pain,—for His sake, in His name, I plead with you for the hospitals this Christmas Day, and for all good works. He that soweth plenteously shall also reap plenteously; or, as it is more forcibly and beautifully put in the original and in our Revised Version, "He that soweth with blessings shall reap with blessings."

[Any reader desiring to give to the Hospitals, is referred to the first three pages of advertisements in this issue. Those who reside in the country cannot do better than send a cheque to their local hospitals, with the names of which they will be familiar.—Ed. T. H.]

